# Chronic kidney disease as a predictive factor for poor prognosis in traumatic brain injury among older adults: a case-control study

**DOI:** 10.3389/fneur.2023.1286592

**Published:** 2023-11-30

**Authors:** Haoyang Mo, Fan Fan, Jian Liu, Wenfan Zhang, Qing Wang, Xiangning Yuan

**Affiliations:** ^1^Department of Neurosurgery, Xiangya Hospital, Central South University, Changsha, Hunan, China; ^2^National Clinical Research Center for Geriatric Disorders, Xiangya Hospital, Central South University, Changsha, Hunan, China; ^3^Department of Medical Record, Xiangya Hospital, Central South University, Changsha, Hunan, China; ^4^Department of Interventional Medicine and Vascular Surgery, Hunan Provincial People's Hospital, The First Affiliated Hospital of Hunan Normal University, Changsha, Hunan, China; ^5^Department of Nephrology, Xiangya Hospital, Central South University, Changsha, Hunan, China

**Keywords:** traumatic brain injury, clinical research, prognosis, chronic kidney disease, glasgow coma scale score, serum creatine

## Abstract

**Objective:**

Traumatic brain injury (TBI) is a highly prevalent neurological disorder that affects a gradually increasing proportion of older adults. Chronic kidney disease (CKD) significantly contributes to global years of life lost, with an estimated one-tenth of the global population affected by CKD. However, it remains unclear whether CKD impacts TBI prognosis. We conducted a case-control study to investigate the clinical outcomes of TBI patients with or without CKD comorbidity and identified the risk factors associated with a poor prognosis.

**Methods:**

From January 2017 through April 2023, 11 patients with TBI and CKD were included, and 27 control TBI cases with normal kidney function were matched by age, gender, and admission Glasgow Coma Scale (GCS) score as the control group.

**Results:**

The CKD TBI group had a significantly lower GCS score upon discharge (7.1 ± 5.9) compared to the non-CKD TBI group (13.1 ± 2.6) (*p* < 0.01). ICU stay time and hospitalization expenses were higher in the CKD group than the non-CKD group, though there were no statistical differences. Additionally, patients in the CKD TBI group had a higher frequency of hospital-acquired infections (54.4%) compared with those in the non-CKD TBI group (7.4%) (*p* < 0.01). The two groups exhibited no differences in hemoglobin levels, albumin levels, or coagulation function. Logistic regression analysis showed that advanced age, low admission GCS score, elevated blood urea, and creatinine levels were associated with a poor neurological prognosis.

**Conclusion:**

TBI patients comorbid with CKD have a poorer prognosis than those with normal kidney function.

## 1 Introduction

Traumatic brain injury (TBI) is a highly prevalent neurological disorder that is impacting a gradually increasing proportion of elderly patients. Falls and road traffic accidents are the leading causes of TBI. The acute injury caused by TBI and the ensuing long-term functional impairments that present themselves during rehabilitation significantly burden patients' health (1–3). Chronic kidney disease (CKD) is a significant contributor to global years of life lost, and an estimated one-tenth of the global population may be affected by CKD. The rising prevalence of chronic conditions such as diabetes, hypertension, and obesity elevates the magnitude of CKD as a pressing public health concern (4–6).

A growing amount of attention is being given to brain-kidney interactions, and some studies have explored the association between TBI and kidney dysfunction, with the majority of the research focusing on the relationship between TBI and post-traumatic acute kidney injury (AKI) (7, 8). Although the mechanisms are not fully understood, potential factors contributing to TBI-induced AKI include post-injury inflammation and the dysregulation of catecholamine release (9). Wu et al. reported that an increased risk of long-term CKD development was associated with TBI (10). However, it remains unclear whether CKD impacts TBI prognosis. Therefore, we conducted a case-control study to investigate the clinical outcomes of older TBI patients comorbid with CKD and identified the risk factors associated with a poor prognosis.

## 2 Methods

This retrospective case-control study did not involve traceable personal information, and patient consent was not required. The ethics committee of Xiangya Hospital, Central South University, approved the study. This study was reported following guidelines written according to the STROBE (Strengthening the Reporting of Observational Studies in Epidemiology) statement (11).

### 2.1 Participants

From January 2017 through April 2023, brain injury patients admitted to Xiangya Hospital of Central South University were included in the trial if they had traumatic brain injury, were aged ≥65 years old, and had CKD [eGFR <60 mL/(min·1.73 m^2^) lasting for at least 3 months].

### 2.2 Data collection

The medical records and relevant examination data of the included patients were retrieved from our medical center's electronic medical record system. The demographic data included the patients' age, gender, admission Glasgow Coma Scale (GCS) score, mechanism of injury, lesions of injuries, and history of pre-existing chronic diseases. After reviewing each patient's computed tomography (CT) scan data, we documented radiological features such as epidural hematoma, subdural hematoma, cerebral contusion, and other relevant findings. The biochemical parameters of the patients mainly included the initial admission levels of hemoglobin (Hb), albumin (Alb), serum urea, serum creatinine (Cr), coagulation function parameters, and etiological culture results.

### 2.3 Outcomes

The primary outcomes included the patients' discharge GCS score, infection status during hospitalization, number of days of hospital stay, number of days of intensive care unit (ICU) stay, and hospitalization expenses. The secondary outcome measure was the difference in discharge and admission GCS scores. If the discharge GCS score was lower than the admission GCS score, the patient was defined as having an unfavorable outcome.

### 2.4 Statistical analysis

Categorical data are presented as counts and percentages, and continuous data are expressed as mean ± standard deviation or median (interquartile range). A comparison between categorical data was performed using the chi-square test, and continuous data were compared using the *t-*test or non-parametric tests.

To investigate the relative risk of potential predictor variables, univariate logistic regression was applied to obtain the odds ratios (ORs) and 95% confidence intervals (CIs). Based on the significant factors identified in the univariate analysis, a receiver operating characteristic (ROC) curve model was established. The model's discriminatory power was evaluated by calculating the area under the curve (AUC) of the ROC curve. The cutoff value was determined by maximizing the Youden index in the ROC curve analysis. A *p-*value of < 0.05 was considered statistically significant. All data analysis was performed using R version 4.1.0.

## 3 Results

### 3.1 Patient inclusion and characteristics

**Figure 1:**
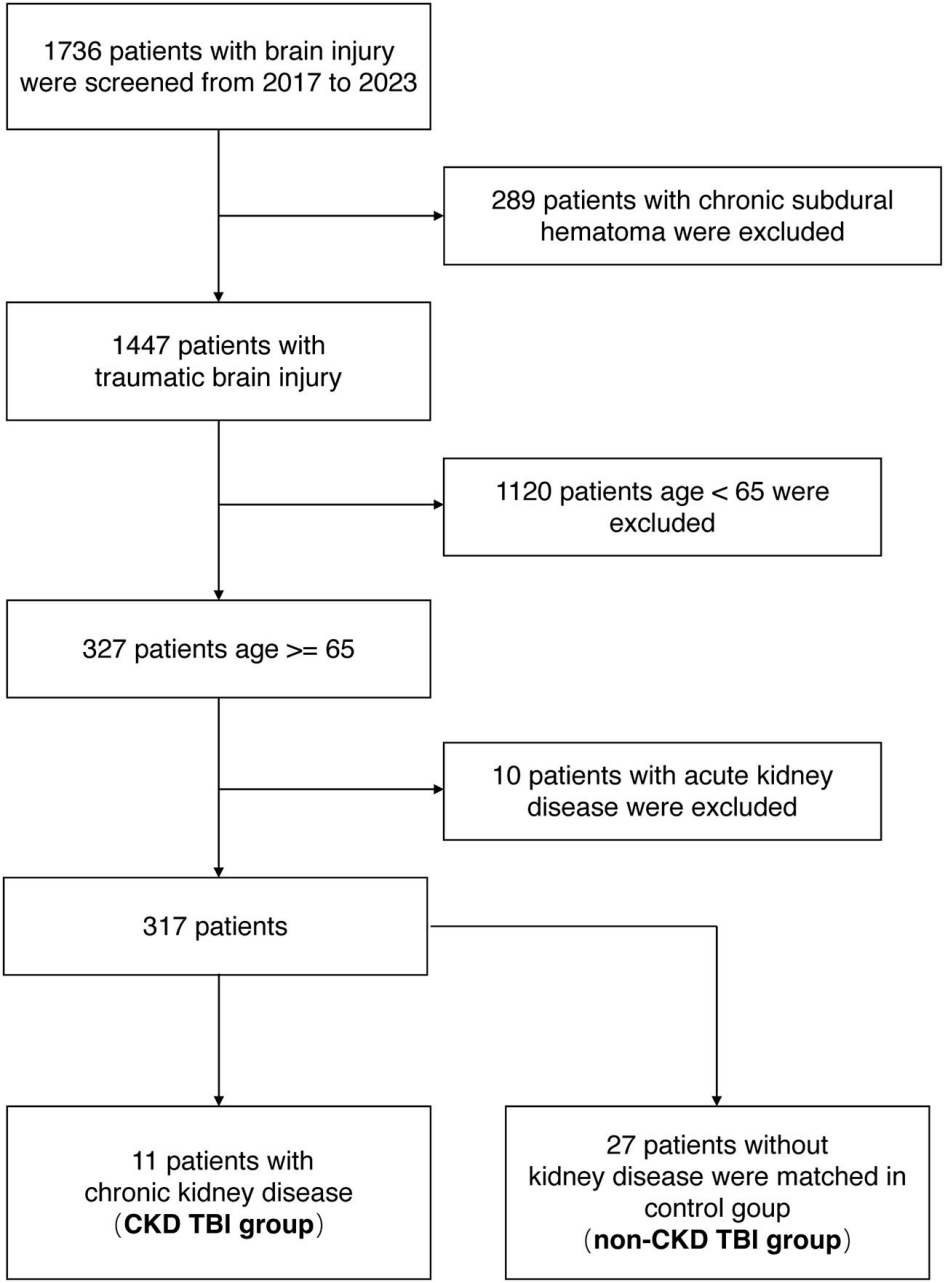
Flowchart for selection of analyzed participants.

From January 2017 through April 2023, 1,736 brain injury patients were admitted to Xiangya Hospital of Central South University. Of these, 289 cases of chronic subdural hematoma, 1,120 cases under the age of 65, and 10 cases of AKI were excluded. Finally, 11 cases of TBI comorbid with CKD were screened (CKD TBI group). A total of 27 TBI patients with normal kidney function were matched for age, gender, and admission GCS scores and included in the control group (non-CKD TBI group) (Figure 1).

A total of 38 TBI patients were analyzed. Table 1 presents their baseline characteristics. The average age of the 38 patients was 70.5 ± 4.5 years, and there were 31 males (81.6%) (Table 1). The GCS score on admission was 11.6 ± 2.7. Falling was the most common mechanism of injury, accounting for 47.4% of all injuries. Other mechanisms included traffic accidents (36.8%) and assaults (5.3%). Imaging examinations revealed intracranial lesions, including epidural hematoma (13.2%), subdural hematoma (55.3%), and cerebral contusion (65.8%) (Table 1).

**Table 1:** Baseline characteristics of included patients.

	**ALL**	**Non-CKD**	**CKD**	***P* value**
	***N** = **38***	***N** = **27***	***N** = **11***	
Age (y)	70.5 (4.5)	69.7 (4.3)	72.5 (4.7)	0.107
**Sex**, ***n*** **(%)**				0.648
Female	7 (18.4)	6 (22.2)	1 (9.1)	
Male	31 (81.6)	21 (77.8)	10 (90.9)	
GCS admission	11.6 (2.7)	12.1 (2.5)	10.5 (3.0)	0.126
**Mechanism**, ***n*** **(%)**				0.938
Traffic accident	14 (36.8)	10 (37.0)	4 (36.4)	
Falling	18 (47.4)	13 (48.1)	5 (45.5)	
Assault	2 (5.3)	1 (3.7)	1 (9.1)	
Other	4 (10.5)	3 (11.1)	1 (9.1)	
**Location of lesions**, ***n*** **(%)**				
EDH	5 (13.2)	4 (14.8)	1 (9.1)	1.000
SDH	21 (55.3)	13 (48.1)	8 (72.7)	0.282
Cerebral contusion	25 (65.8)	17 (63.0)	8 (72.7)	0.714
**Medical history**, ***n*** **(%)**				
Hypertension	21 (55.3)	11 (40.7)	10 (90.9)	0.010
Diabetes	6 (15.8)	1 (3.7)	5 (45.5)	0.005
CHD	10 (26.3)	3 (11.1)	7 (63.6)	0.002
Stroke	2 (5.3)	2 (7.4)	0 (0.0)	1.000
Hb (g/L)	108.8 (22.2)	110.2 (22.9)	105.2 (20.7)	0.517
Alb (g/L)	32.9 (5.7)	33.2 (6.0)	32.3 (5.1)	0.645
Urea (mmol/L)	8.8 (8.4)	5.0 (1.4)	18.2 (10.9)	0.002
Cr (μmol/L)	142.8 (148.2)	75.9 (13.6)	307.1 (197.5)	0.003
**PT**, ***n*** **(%)**				0.295
Long	5 (13.5)	5 (19.2)	0 (0.0)	
Normal	32 (86.5)	21 (80.8)	11 (100.0)	
**APTT**, ***n*** **(%)**				0.540
Long	3 (8.1)	3 (11.5)	0 (0.0)	
Normal	34 (91.9)	23 (88.5)	11 (100.0)	

CKD, chronic kidney disease; GCS, glasgow coma scale; EDH, epidural hematoma; SDH, subdural hematoma; CHD, coronary heart disease; Hb, hemoglobin; Alb, albumin; Cr, creatinine; PT, prothrombin time; APTT, activated partial thromboplastin time.

Patients in the CKD TBI group had a higher frequency of comorbidities such as hypertension, diabetes, and coronary heart disease (*p* < 0.05) (Table 1). We also recorded detailed laboratory markers upon the patients' admission. The average values for Hb and Alb were 108.8 g/L and 32.9 g/L, respectively. The patients in the CKD TBI group had lower levels of Hb and Alb compared to the non-CKD TBI group, although the differences were not statistically significant. There was no difference in coagulation function between the two groups. Regarding renal function markers, patients from the CKD TBI group showed significantly higher levels of serum urea (18.2 ± 10.9 mmol/L) and serum creatinine (307.1 ± 197.5 μmol/L) than patients from the non-CKD TBI group, with values of 5.0 ± 1.4 mmol/L and 75.9 ± 13.6 μmol/L, respectively (*p* < 0.01) (Table 1).

### 3.2 Clinical outcomes

Next, we compared the overall outcomes of the two patient groups upon discharge (Table 2). It is worth noting that the patients from the CKD TBI group had a significantly lower GCS scores upon discharge (7.1 ± 5.9) compared with the non-CKD TBI group (13.1 ± 2.6) (*p* < 0.01). The median length of hospital stay was comparable between the two groups. Patients in the CKD TBI group spent longer in the ICU, and their overall hospitalization costs were higher than those of the non-CKD TBI group. However, the differences were not statistically significant.

**Table 2:** Comparison of clinical outcomes between TBI patients with CKD and without CKD.

	**ALL *N = 38***	**Non-CKD *N = 27***	**CKD *N = 11***	***P* value**
Hospital stay (days)	14.0 (9.0, 19.8)	14.0 (9.5, 18.5)	13.0 (7.5, 29.0)	0.847
ICU stay (days)	3.0 (0.0, 12.8)	3.0 (0.0, 9.5)	10.0 (1.0, 29.0)	0.115
Cost (¥)	113,358.9 (43,541.0, 154,290.8)	110,152.7 (39,286.9, 132,064.2)	146,633.3 (48,707.2, 361,544.5)	0.111
GCS discharge	11.4 (4.7)	13.1 (2.6)	7.1 (5.9)	0.007
**Infection**, ***n*** **(%)**				0.004
No	30 (78.9)	25 (92.6)	5 (45.5)	
Yes	8 (21.1)	2 (7.4)	6 (54.5)	

CKD, chronic kidney disease; ICU, intensive care unit; GCS, glasgow coma scale.

Additionally, patients in the CKD TBI group had a higher frequency of hospital-acquired infections (54.4%) compared with the non-CKD TBI group (7.4%) (*p* < 0.01) (Table 2). Four patients in the CKD TBI group experienced multi-species and multi-site infections with various pathogens, including gram-negative bacteria, gram-negative cocci, and fungi. The most common site of infection was the lungs, followed by the urinary tract and central nervous system.

**Figure 2:**
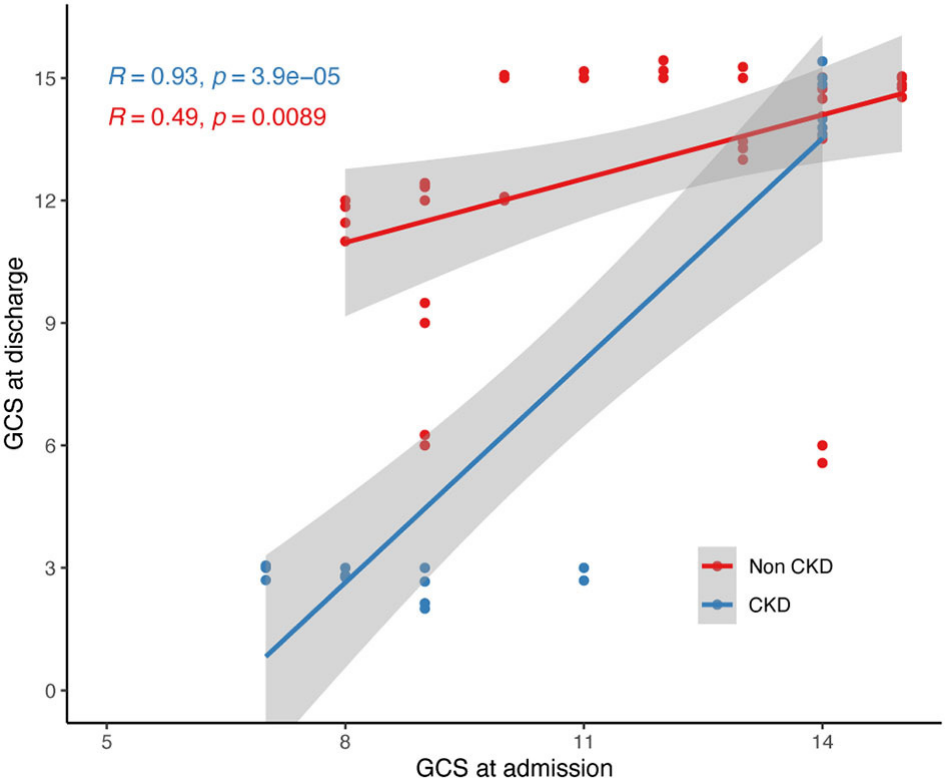
Linear regression of admission and discharge GCS scores of two group of participants. GCS, glasgow coma scale.

Furthermore, we conducted linear regression analysis on both groups' admission and discharge GCS scores (Figure 2). The CKD TBI group showed a fitted curve with an R-value of 0.93 (*p* < 0.001), while the non-CKD TBI group had a fitted curve with an R-value of 0.49 (*p* < 0.01). Among the TBI patients with lower admission GCS scores, those with comorbid CKD had lower GCS scores upon discharge than those without CKD. Conversely, for patients in both groups with higher admission GCS scores, higher GCS scores were observed upon discharge.

### 3.3 Association of renal indicators with outcomes

After defining a discharge GCS score lower than the admission GCS score as a indicator of an unfavorable outcome, we calculated the ORs for the relevant indicators in univariate logistic models for adverse prognostic outcomes (Table 3). Advanced age upon admission, low admission GCS score, and high levels of initial serum urea and serum creatinine were closely associated with poor TBI outcomes.

**Table 3:** Univariate logistic regression for independent variables related to poor outcomes.

	**OR (95% CI)**	***P* value**
Age	1.30 (1.06, 1.60)	0.013
GCS admission	0.56 (0.37, 0.85)	0.006
Urea	1.47 (1.12, 1.93)	0.006
Cr	1.01 (1.00, 1.02)	0.017

OR, odds ratio; GCS, glasgow coma scale; Cr, creatinine.

**Figure 3:**
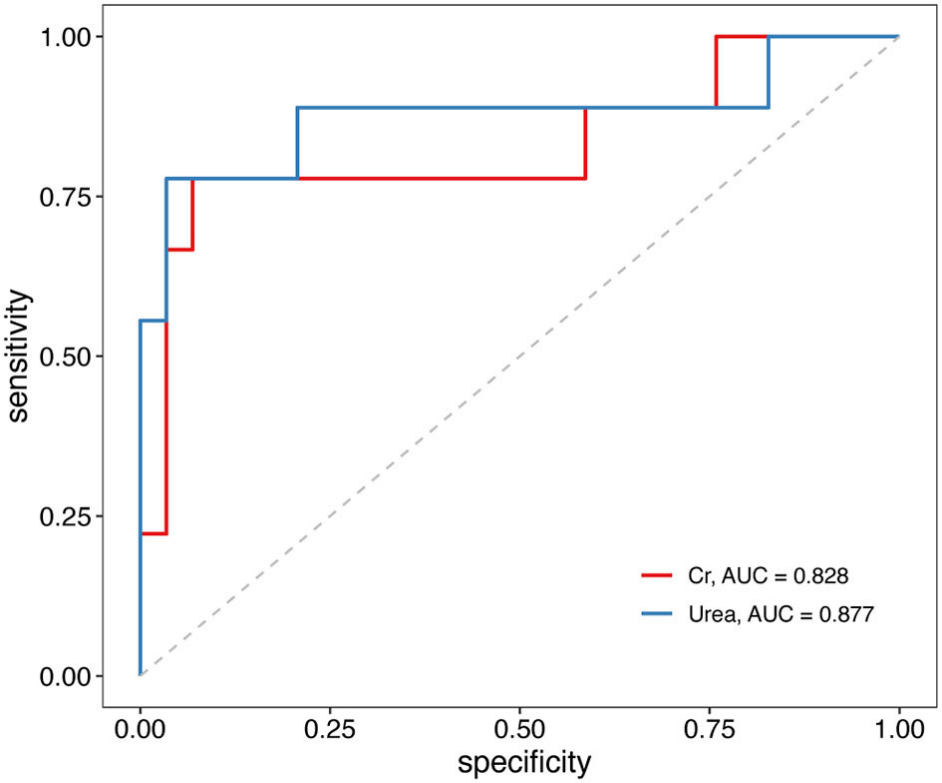
Receiver operating characteristic (ROC) curves of serum urea and creatinine associations with unfavorable outcomes in TBI patients. TBI, traumatic brain injury.

Next, we constructed ROC curves to explore the ability of serum urea and serum creatinine to predict adverse outcomes in TBI (Figure 3). The results indicated that serum urea and serum creatinine can be used to predict poor prognostic outcomes in TBI, with AUCs of 0.877 and 0.828, respectively. The optimal cutoff values for serum urea and creatinine were 10.97 (mmol/L) and 129.50 (μmol/L), respectively.

## 4 Discussion

Aging is a global problem, and TBI has become increasingly prevalent among older adults (12). With the increase in the prevalence of chronic diseases such as diabetes in adults, the burden of renal failure is also increasing (13). This case-control study analyzed the clinical outcomes and risk factors of TBI patients with CKD comorbidity. Among cases with similar severity of brain injury, TBI patients with concurrent CKD generally exhibited poorer outcomes compared to the general TBI population.

TBI patients with concurrent CKD constitute a high-risk population in neurointensive care and require special attention from treating physicians. Our study identified urea and creatinine as serum biomarkers that can be used to predict adverse outcomes in TBI events. As renal function progressively declines, CKD patients may experience various complications such as anemia, malnutrition, and coagulation dysfunction (6). However, when we compared hemoglobin levels, serum albumin levels, and coagulation function indexes between the CKD TBI group and the non-CKD TBI group in this study, there were no statistical differences between the two groups, and logistic regression did not indicate that these indexes affect the prognosis of TBI patients.

This study found that patients with TBI comorbid with CKD had a higher prevalence of chronic diseases, such as hypertension, diabetes, and coronary heart disease. These diseases can cause arteriosclerosis or worse vascular conditions, which may be related to a worse prognosis for TBI patients with CKD (14, 15). In addition, CKD patients tend to have internal environment disorders such as azotemia. The accumulation of various metabolic substances in CKD may exacerbate the severity of brain injury in TBI patients (16, 17). For instance, guanidine compounds, a group of uremic neurotoxins, have neurotoxic and vascular toxic effects on neurons and blood vessels (18). Another uremic neurotoxin, asymmetric dimethylarginine, may affect cerebral blood flow and decrease cerebral perfusion (19). Azotemia in CKD patients can also exacerbate brain injury, which manifests as an aggravation of post-traumatic brain edema by disrupting the blood-brain barrier via osmotic pressure disturbances.

CKD patients are prone to infection. As CKD is a systemic disease, prolonged energy insufficiency, the accumulation of various inflammatory mediators, and the dysregulation of the autonomic nervous system may attenuate immunity in patients (20, 21). In this study, we observed a significantly higher incidence of infection in TBI patients with comorbid CKD compared to the non-CKD group. Moreover, multiple pathogens and multiple site infections were more common in TBI patients comorbid with CKD and may lead to a prolonged ICU stay and increased hospitalization costs. Therefore, doctors need to monitor for infection in TBI patients comorbid with CKD and be alert to hospital-acquired infections.

There were several limitations in our study. Firstly, this study was inherently a single-center retrospective case-control study with a relatively small number of patients included, which may restrict the generalizability of our conclusions. Therefore, caution should be exercised when extending our findings to a broader population. Further validation of our findings is warranted through prospective studies based on larger cohorts. Secondly, due to limitations in the original medical records data, we used the discharge GCS score as the outcome measure, but data on the long-term prognosis of patients after discharge should be collected as further evaluation criteria. Thirdly, it is worth noting that other underlying comorbidities in the elderly population may act as confounding factors that influence the interpretation of our results. Future research should consider incorporating additional relevant factors in the case matching process.

In summary, TBI patients comorbid with CKD have a poorer prognosis than those with normal kidney function. Elevated serum urea and creatine levels are considered risk factors for poorer clinical prognosis in older TBI patients.

## Data availability statement

The original contributions presented in the study are included in the article/supplementary material, further inquiries can be directed to the corresponding author.

## Ethics statement

The studies involving humans were approved by the Ethics Committee of Xiangya Hospital, Central South University. The studies were conducted in accordance with the local legislation and institutional requirements. Written informed consent for participation was not required for this study in accordance with the national legislation and the institutional requirements.

## Author contributions

HM: Data curation, Formal analysis, Funding acquisition, Visualization, Writing – original draft. FF: Data curation, Formal analysis, Project administration, Resources, Writing – review & editing. JL: Data curation, Formal analysis, Writing – review & editing. WZ: Data curation, Formal analysis, Writing – review & editing. QW: Writing – review & editing. XY: Conceptualization, Funding acquisition, Resources, Supervision, Writing – review & editing.
